# *In vitro *evaluation of a double-stranded self-complementary adeno-associated virus type2 vector in bone marrow stromal cells for bone healing

**DOI:** 10.1186/1479-0556-9-4

**Published:** 2011-02-27

**Authors:** Farhang Alaee, Osamu Sugiyama, Mandeep S Virk, Ying Tang, Bing Wang, Jay R Lieberman

**Affiliations:** 1 New England Musculoskeletal Institute, Department of Orthopaedic Surgery, University of Connecticut Health Center, 263 Farmington Avenue, Farmington, CT, 06030, USA; 2 Department of Orthopaedic Surgery, University of Pittsburgh, Pittsburgh, PA, 15219, USA

## Abstract

**Background:**

Both adenoviral and lentiviral vectors have been successfully used to induce bone repair by over-expression of human bone morphogenetic protein 2 (BMP-2) in primary rat bone marrow stromal cells in pre-clinical models of *ex vivo *regional gene therapy. Despite being a very efficient means of gene delivery, there are potential safety concerns that may limit the adaptation of these viral vectors for clinical use in humans. Recombinant adeno-associated viral (rAAV) vector is a promising viral vector without known pathogenicity in humans and has the potential to be an effective gene delivery vehicle to enhance bone repair. In this study, we investigated gene transfer in rat and human bone marrow stromal cells in order to evaluate the effectiveness of the self-complementary AAV vector (scAAV) system, which has higher efficiency than the single-stranded AAV vector (ssAAV) due to its unique viral genome that bypasses the rate-limiting conversion step necessary in ssAAV.

**Methods:**

Self-complementaryAAV2 encoding GFP and BMP-2 (scAAV2-GFP and scAAV2-BMP-2) were used to transduce human and rat bone marrow stromal cells *in vitro*, and subsequently the levels of GFP and BMP-2 expression were assessed 48 hours after treatment. In parallel experiments, adenoviral and lentiviral vector mediated over-expression of GFP and BMP-2 were used for comparison.

**Results:**

Our results demonstrate that the scAAV2 is not capable of inducing significant transgene expression in human and rat bone marrow stromal cells, which may be associated with its unique tropism.

**Conclusions:**

In developing e*x vivo *gene therapy regimens, the ability of a vector to induce the appropriate level of transgene expression needs to be evaluated for each cell type and vector used.

## Background

The healing of large bone defects presents a challenge for regenerative medicine. Autologous bone grafting is the current gold standard to promote bone repair, but in many cases there is insufficient amounts of autologous bone graft available to heal the defect. In addition, there is morbidity associated with bone graft harvest [1].

Recombinant human BMP-2 (rhBMP2) and BMP-7 (rhBMP7) are two osteoinductive agents that are presently available for clinical use. RhBMP-2 is FDA approved for use in anterior spinal fusion and open tibial shaft fractures [2]. RhBMP-7 (OP-1) was shown to have comparable efficacy to autologous bone grafting in the treatment of tibial non-unions without the donor site morbidity [3]. Nevertheless, these recombinant proteins are expensive and require supraphysiologic doses to achieve the desired clinical effect [4]; there are also concerns that these high doses are associated with side effects such as soft tissue edema or heterotopic bone formation [5,6]. Therefore, there has been interest in developing gene therapy as a strategy to deliver proteins to a specific bone repair site, particularly in cases where there are large bone defects or defects associated with severe soft tissue injury.

The use of viral vectors that over-express BMP-2 has been successful in promoting bone repair in a variety of pre-clinical animal models of bone defect healing [7-10]. In previous studies of *ex vivo *gene therapy in our laboratory, lentiviral and adenoviral mediated over-expression of BMP-2 in rat bone marrow stromal cells successfully healed a critical sized rat femoral defect [11-13]. Overexpression of BMP-2 by lentiviral transduction induced superior quality of bone repair compared to adenoviral transduced cells as noted in biomechanical testing and u-CT bone volumetric data [13].

Safety is a critical issue in identifying the appropriate viral vector for human use. Since gene therapy for bone repair would be used to treat a non-lethal condition, any increase in morbidity or mortality would not be acceptable. Insertional mutagenesis and emergence of replication competent viral particles remain areas of concern with respect to lentiviral vectors and the safety of these vectors needs to be evaluated in human trials [14,15]. The adenoviral vector does not integrate into the host chromosomes and the potential risk of insertional mutagenesis is less than the lentiviral vectors [16]. However, adenoviral vectors induce strong cellular and humoral immune responses in the host that results in tissue injury and loss of transgene expression [16-18].

Recombinant adeno-associated viral (AAV) vector is a small non-enveloped single-stranded DNA virus [19]. This unique viral vector has the distinct advantage of being capable of infecting a wide range of host cell types including dividing and non-dividing cells [20]. In addition, there is no conclusive evidence indicating pathogenicity of AAV vector in humans [21]. AAV also induces long-term gene expression in transduced cells and its persistence is thought to be mostly extra-chromosomal [16,21]. The lower risk of random genomic integration of AAV in comparison with other viral vectors is considered to be a safety advantage [21,22]. Several studies have also shown very low cell-mediated immunogenicity of AAV that could facilitate the long-term expression of the transgene [23-25]. A number of AAV serotypes have been used as delivery methods in gene therapy and each serotype has a distinct affinity for certain cell types that is because of differences in cell binding and/or intracellular trafficking [21]. AAV2 is the most widely used serotype in human clinical trials and has a broad range of tissue tropism in several species [21]. A number of clinical trials have been approved by the FDA to assess AAV2 in treatment of a variety of human diseases including inflammatory arthritis, cystic fibrosis, alpha-1 antitrypsin deficiency, epilepsy, hemophilia B, Parkinson's disease and muscular dystrophies [26]. The aim of this study was to evaluate the efficacy of a self-complementary AAV2 vector system in transducing human and rat bone marrow stromal cells in comparison with lentiviral and adenoviral vectors.

## Methods

### Viral Vector Production

**Figure 1:**
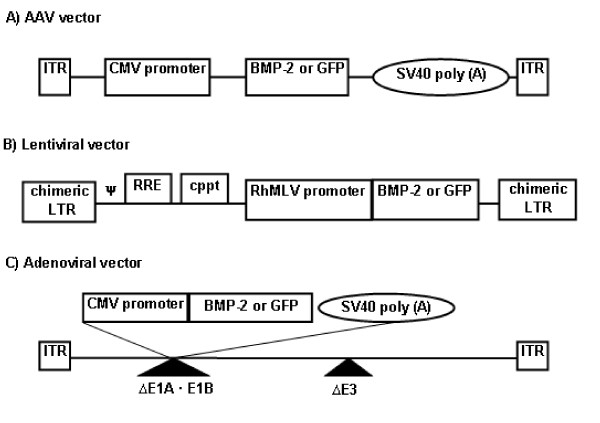
**Schematic Drawings of the Viral Vectors **A) AAV vector that consists of inverted terminal repeats (ITR) at 3' and 5' ends with BMP-2 or GFP under the control of CMV promoter and SV40 poly(A), B) Lentiviral vector that consists of long terminal repeats (LTR) with RhMLV promoter driving the expression of BMP-2 or GFP and C) Adenoviral vector that consists of ITRs and BMP-2 or GFP under the control of CMV promoter as well as SV40 poly(A). The adenoviral vector carries deletions in E1 and E3 regions rendering it replication deficient except in 293 cell lines (including 293T cells) that are capable of substituting E1 function, hence the toxicity of this vector to 293T cells. ψ: packaging signal, cppt: central polypurine tract, RRE: Rev-responsible element, SV40 poly(A): simian virus poly adenilation signal sequence

AAV plasmids (double-stranded, serotype 2) encoding rhBMP-2 and enhanced GFP (eGFP) cDNA under CMV promoter were constructed, respectively (Figure 1A). The serotype 2 of AAV viruses (AAV-BMP-2 and AAV-eGFP) were produced according to the method previously described [27]. The AAV particles were purified by CsCl density gradient ultracentrifugation. The AAV viral genomes were quantified by DNA dot blot and were in the range of 1 × 10^12 ^to 5 × 10^12 ^viral genomes/ml according to a previously published protocol [28]. Lentiviral vectors encoding BMP-2 and eGFP cDNA under RhMLV promoter (LV-BMP-2 and LV-GFP) were generated by calcium phosphate-mediated co-transfection of plasmids in 293T cells as previously described (Figure 1B) [29]. Lentiviral particles were concentrated 100-fold by ultracentrifugation. The titer of eGFP-expressing lentiviral vector was determined by infection of 293T cells, followed by flow cytometric analysis of the percentages of eGFP-positive cells. The titer of BMP-2-expressing vector was estimated by comparison of p24 levels with eGFP-expressing vector. The titers of lentiviral vectors were in the range of 1 × 10^8 ^to 3 × 10^8 ^transducing units/ml.

Adenoviral vectors encoding BMP-2 and eGFP cDNA under the CMV promoter (Ad-BMP-2 and Ad-GFP) were prepared as previously described (Figure 1C) [30]. Adenoviral vectors were propagated in 293 cells and cell lysates were concentrated by CsCl density gradient ultracentrifugation. Viral stocks were subsequently purified by dialysis in phosphate buffered saline (PBS) (Invitrogen, Carlsbad, CA, USA) containing 10% glycerol. The titers of adenoviral vectors were in the range of 5 × 10^9 ^to 1 × 10^10 ^transducing units/ml.

### Cell preparation and transduction with AAV, lentiviral and adenoviral vectors

Institutional approval for the use of rats to harvest bone marrow cells was obtained from the University of Connecticut Health Center animal care committee. The bone marrow cells were harvested from 8 week-old male Lewis rats (Charles Rivers Labs Inc. Wilmington, MA, USA). The rats were euthanized using CO2 asphyxiation and the primary rat bone marrow cells were collected from the femurs and the tibias by flushing the medullary canal with Iscove's Modified Dulbecco's Medium (IMDM) (Invitrogen, Carlsbad, CA, USA). The cells were maintained in IMDM containing 15% fetal bovine serum (FBS) (Omega Scientific, Tarzana, CA, USA), 100 U/ml penicillin and 100 mg/ml streptomycin sulfate until they reached passage three.

Human bone marrow stromal cells which were CD45 negative and CD90, CD105 and CD146 positive [31] (Gift of Pamela Robey, National Institute of Dental and Craniofacial Research, Bethesda, MD. USA) were maintained in Minimum Essential Medium (MEM) Alpha Medium (Invitrogen, Carlsbad, CA, USA) containing 20% FBS, 2 mM L-glutamine, 100 U/ml penicillin and 100 mg/ml streptomycin sulfate.

One million rat and human bone marrow stromal cells and 293T cells (ATCC, Manassas, VA, USA) were transduced with LV-BMP-2 or LV-GFP in the presence of polybrene for 24 hours at MOI of 25. Transduction of one million rat and human bone marrow stromal cells and 293T cells with AAV-BMP-2 and AAV-GFP was carried out using10^5 ^viral genomes/cell in serum free media for 1 hour and another 23 hours in regular media. One million rat and human bone marrow stromal cells and 293T cells (ATCC, Manassas, VA, USA) were transduced with Ad-BMP-2 or Ad-GFP in serum free media for 4 hours at MOI of 100, which was then replaced by regular media and maintained for 20 more hours. In all transduction protocols, the culture media was replaced by fresh media after the first 24 hours and the assessment of BMP-2 production or eGFP expression was carried out 24 hours after the addition of the fresh media. Since transduction with each viral vector was done at the specified MOI and 1 million cells were used in all experiments, the number of viral particles of each vector was the same in the experiments.

### ELISA for BMP-2

In vitro BMP-2 production induced by AAV-BMP-2, Ad-BMP-2 or LV-BMP-2 transduced cells during a 24-hour period was quantified by a commercial enzyme linked immunosorbent assay (ELISA) kit (Quantikine, R&D, Minneapolis, MN, USA) according to the manufacturer's protocol. Briefly, one million rat bone marrow stromal cells or 293T cells were plated into 10-cm culture dishes. 24 hours after transduction the medium was replaced by 10 ml of fresh medium. Cells were incubated for another 24 hours after which culture supernatants were harvested for BMP-2 measurement. The BMP-2 production was measured in triplicate and reported as nanograms of BMP-2/one million cells/24 h.

### Fluorescent Microscopy

The visualized GFP expression in transduced cells was detected under fluorescent microscopy (Nikon Eclipse TE2000-U, Nikon Instruments Inc., Melville, NY, USA) at two days after transduction. The cell images were taken by SPOT advanced software (Diagnostic Instruments, Inc., Sterling Heights, MI, USA).

### Statistical Analysis

Student t-test was used to compare BMP-2 levels induced by viral vectors in human and rat bone marrow stromal cells and 293T cells. P values less than 0.05 were considered significant.

## Results

### GFP expression in the human and rat bone marrow stromal cells transduced with AAV2-GFP, LV-GFP and Ad-GFP

293T cells were used as a control for GFP expression in transduced human and rat bone marrow stromal cells.

**Figure 2:**
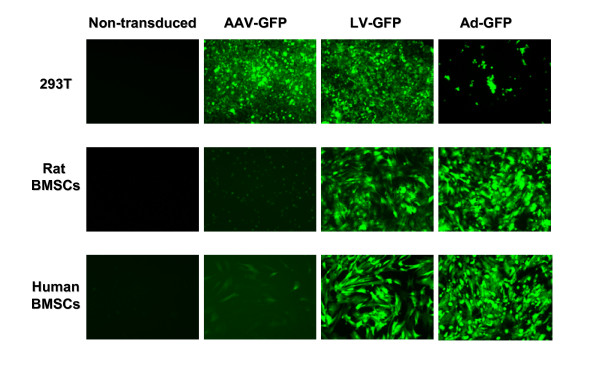
**GFP Expression in Viral Transduced Cells **1 million rat and human bone marrow stromal cells (BMSCs) and 293T cells were transduced with AAV-GFP, LV-GFP and Ad-GFP and the GFP expression was assessed 48 hours after transduction. Non-transduced cells served as negative control. In comparison with the non-transduced controls, AAV-GFP transduced 293T cells show strong eGFP expression, but rat and human bone marrow stromal cells (BMSCs) did not exhibit expression levels as strong as 293T cells. LV-GFP and Ad-GFP transduced cells showed strong GFP expression in all transduced cell types except for Ad-GFP transduced 293T cells in which the viral replication causes cell death.

The scAAV2-GFP showed strong GFP expression in transduced 293T cells 48 hours post-infection, but in human and rat bone marrow stromal cells it did not show GFP expression as strongly as transduced 293T cells at the same time point. In contrast, strong GFP expression was detectable in all three cell types transduced with LV-GFP. Ad-GFP transduced human and rat bone marrow stromal cells showed strong GFP expression, too. Although the surviving Ad-GFP transduced 293T cells did show strong levels of GFP expression, the majority of the adenoviral transduced 293T cells had died by 48 hours secondary to the adenoviral toxicity to these cells (Figure 2).

### BMP-2 production by the rat bone marrow stromal cells transduced with the AAV-BMP-2, LV-BMP-2 and Ad-BMP-2

BMP-2 production in 1 million transduced 293T or rat bone marrow stromal cells was quantified by ELISA using the culture supernatants harvested 24 hours after addition of fresh medium.

**Figure 3:**
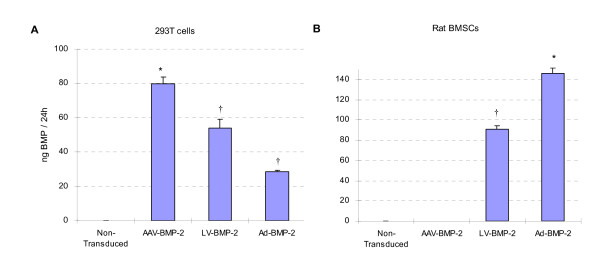
**BMP-2 Production by Viral Transduced Cells **1 million rat bone marrow stromal cells (BMSCs) and 293T cells were transduced with AAV-BMP-2, LV-BMP-2 and Ad-BMP-2 and the BMP-2 production was quantified 48 hours after transduction. Non-transduced cells served as negative control. A) Amongst the three viral vectors used to over express BMP-2, AAV-BMP-2 induced the highest amount of BMP-2 production in 293T cells. Transducing the 293T cells with Ad-BMP-2 induced cell death after 24 hours in culture. B) AAV-BMP-2 transduced rat bone marrow stromal cells (BMSCs) did not produce any detectable amount of BMP-2 as opposed to LV-BMP-2 and Ad-BMP-2 transduced rat bone marrow stromal cells which made significantly higher amounts of BMP-2.*: P value < 0.05 compared to all other groups, †: P value < 0.05 compared to non-transduced group

In 293T cells which were used as a control cell line, BMP-2 production was induced by all three of the viral vectors. BMP-2 levels were approximately 47% higher in 293T cells transduced with AAV-BMP-2 (79.61 ± 4.14 ng) compared to those transduced with LV-BMP-2 (53.96 ± 5.21 ng), (P < 0.05). BMP-2 production by Ad-BMP-2 transduced 293T cells (28.59 ± 0.64 ng) showed a dramatic decrease and was only 35% of the levels achieved by AAV group. The low BMP-2 production by adenoviral transduced cells was due to the fact that adenoviral particles are known to be toxic to 293T cells (Figure 3A).

In rat bone marrow stromal cells, Ad-BMP-2 and LV-BMP-2 induced high levels of BMP-2 production (146.1 ± 5.1 ng and 90.8 ± 3.2 ng, respectively) whereas AAV-BMP-2 did not produce any detectable amount of BMP-2 production at 10^5 ^viral genomes/cell, (P < 0.05) (Figure 3B).

Collectively, the self-complementary AAV2 system used in these experiments was not capable of inducing adequate levels of gene expression in either the rat or human bone marrow cells in comparison to the lentiviral and adenoviral vector systems.

## Discussion

In this study we performed a comparison between three vector systems (LV, AV, and AAV) that are commonly used in gene therapy approaches to over express BMP-2 or GFP in human and rat bone marrow stromal cells. We used a scAAV2 vector in our experiments and sought to determine its potential utility in our *ex vivo *gene therapy approach for bone repair. The results of the present study showed that scAAV2 produced significantly less BMP-2 in rat bone marrow stromal cells compared to lentiviral and adenoviral vectors. In contrast to the observation by Pagnotto et al [32] in which scAAV2 in human bone marrow mesenchymal stem cells showed high efficiency of gene transfer, the level of GFP expression in human bone marrow stromal cells in our study was not as strong as lentiviral and adenoviral transduced cells after 48 hours of transduction. These results suggest that careful screening of transgene expression by different viral vectors in different cell types is necessary to develop successful strategies for gene therapy.

Different AAV serotypes have been used to transduce different cell types [20,33,34]. Goodrich et al [35] screened serotypes1-6 and 8 of scAAV vectors and showed superior capacity of scAAV2 to induce gene expression in equine chondrocytes and synoviocytes. Wang et al [36] reported successful gene delivery using AAV serotype 8 into cardiac and skeletal muscle cells of mice and hamsters. In contrast, AAV serotype 2 was not capable of transducing these cell types efficiently due to its unique tropism.

In a mouse model of gene therapy for hemophilia B, the use of AAV8 and AAV9 resulted in a much higher expression levels of Factor IX compared with lentiviral gene delivery to hepatocytes [37]. Our results demonstrate higher BMP-2 expression by AAV-BMP-2 in 293T cells compared to the other viral vectors. This shows the usefulness of the AAV vector system if the appropriate target cells can be efficiently transduced.

Several investigators have successfully used AAV vectors to transduce human cells such as human islet cells, CD34 positive peripheral blood progenitor cells and mesenchymal stromal cells [38-40]. Lattermann et al [41] indicated cell-type specific tropism for AAV vector in an experiment where human nucleus polposus (hNP) cells, synovial fibroblasts and bone marrow derived cells were transduced with ssAAV-Luc and Ad-Luc. AAV transduced bone marrow-derived cells and synovial fibroblasts showed significantly lower luciferase expression compared to hNP cells. In contrast, when Ad-Luc was used, human bone marrow derived cells had comparable luciferase expression to hNP cells. Although we used scAAV2 that has shown a higher efficiency compared to ssAAV vector, our results with AAV-GFP and Ad-GFP transduction of human bone marrow cells were similar to this study.

The genome of single stranded AAV vector has to be converted to double-stranded replicative form once it has entered the target cells, which is a rate limiting step in the replicative cycle of AAV [42,43]. Double-stranded, self-complementary AAV vectors bypass this step and provide the opportunity to achieve more efficient transduction [44]. McMahon et al [45] reported that rat bone marrow cells were unable to produce high levels of GFP when transduced with different titers of single-stranded AAV (ssAAV) serotypes 1,2,4,5 and 6. AAV2 still had the highest efficiency compared to the other serotypes. Thus, we hypothesized that a self-complementary double-stranded AAV2 would have higher transduction efficiency for the human and rat bone marrow stromal cells. Nevertheless, our results with double-stranded AAV-BMP-2 and AAV-GFP also showed the relative inefficiency of the scAAV2 in transducing human and rat bone marrow stromal cells. Our speculation is that low transduction efficiency may be due to a scarcity of cell surface receptors for AAV in this particular cell type or a defect in nuclear trafficking of the vector sequence.

Membrane-associated heparan sulfate proteoglycan serves as the primary cell receptor for AAV type 2 [46]. This accounts for the broad host range of AAV vector. Cross-packaging the AAV2 genome into several AAV serotypes has revealed that the viral tropism to different cell lines in rodents, monkeys and humans could be related to their affinity for heparan sulfate [47]. These reports further support our observations that rat bone marrow cells may be resistant to transduction by AAV2 due to lack of certain AAV receptors.

Testing MOIs of 1, 10, 100, 1000 and 10,000, Stender et al [48] found the MOI of 10^4 ^to result in the highest transgene expression of AAV2-eGFP transduced human bone marrow derived mesenchymal stem cells, but reported severely restricted expression levels compared to 293 cells. Selection of the optimum MOI for transduction of rat bone marrow stromal cells with lentiviral and adenoviral vectors and AAV for transduction of human bone marrow stromal cells was based on the previously published articles in our lab and elsewhere [12,29,32]. As for the AAV, an additional experiment was done using 10^4 ^viral genomes/cell of AAV-BMP-2 in rat bone marrow stromal cells with results similar to the higher MOI of 10^5 ^viral genomes/cell (data not shown). However, the possibility of a different MOI being more effective can not be ruled out.

Striated muscle cells are known to be effectively transduced by the AAV vectors [36]. Direct injection of a doxycycline controllable AAV-BMP-2 vector [49] into the hind limb muscle of mice was reported to induce ectopic bone formation likely due to transduction of the muscle cells. In addition, injection of the vector into a CD1 nude mouse calvarial defect loaded with human MSCs demonstrated some bone formation in the defect site, but not complete bone healing. Other reports of coating structural allografts with various AAV vectors [50-52] have indicated success in allograft integration and bone healing in mice via increased vascularization and remodelling (rAAV-RANKL and rAAV-VEGF) and increased bone formation (rAAV-caALK2 and rAAV2.5-BMP-2). The authors hypothesized that a mixed population of cells including MSCs, inflammatory cells and osteoblasts were the potential local cell targets for the AAV vector. Kang et al [53] reported *in vitro *and *in vivo *bone formation using human adipose-derived mesenchymal stem cells transduced with ssAAV2 to over express BMP-7. These studies also highlight the impact of cell type on the development of a successful gene therapy strategy using AAV to promote bone repair.

AAV transduction efficiency in fibroblasts has been shown to be species dependent [54] and the underlying mechanism for inefficient transduction of murine fibroblasts is thought to be due to impaired trafficking into the nuclei of the transduced cells [55]. These reports show that more extensive efforts are needed to optimize the AAV vector for rat and human bone marrow stromal cell transduction by modifying the viral envelope or the steps involved in nuclear trafficking.

## Conclusions

In summary, our data showed that the serotype 2 of self-complementary AAV vector system was unable to induce sufficient levels of transgene expression in both human and rat bone marrow stromal cells. To our knowledge this is the first report on BMP-2 production by a scAAV vector system in primary rat bone marrow stromal cells. Our results demonstrate the influence of cell type on the potential efficacy of different gene therapy strategies that can be used for bone repair and highlights the need for further experiments to understand and overcome the barriers of transduction with AAV in human and rat bone marrow stromal cells.

## Competing interests

The authors declare that they have no competing interests.

## Authors' contributions

All authors have read and approved the final manuscript. FA has interpreted the data and written the manuscript, OS has performed the in-vitro experiments, MSV has helped with the experiments and data interpretation, YT has made the AAV vectors, BW has edited the manuscript and provided the AAV vector and JRL has designed the experiments, interpreted the results and edited the manuscript.
